# γ-secretase inhibitors augment efficacy of BCMA-targeting bispecific antibodies against multiple myeloma cells without impairing T-cell activation and differentiation

**DOI:** 10.1038/s41408-022-00716-3

**Published:** 2022-08-16

**Authors:** Hailin Chen, Tengteng Yu, Liang Lin, Lijie Xing, Shih-Feng Cho, Kenneth Wen, Kimberly Aardalen, Adwait Oka, Joni Lam, Mike Daley, Haihui Lu, Nikhil Munshi, Kenneth C. Anderson, Yu-Tzu Tai

**Affiliations:** 1 Jerome Lipper Multiple Myeloma Center, LeBow Institute for Myeloma Therapeutics, Dana-Farber Cancer Institute, Harvard Medical School, Boston, MA USA; 2 Hematology Department, Yueyang Hospital of Integrated Traditional Chinese and Western Medicine, Shanghai University of Traditional Chinese Medicine, Shanghai, China; 3 State Key Laboratory of Experimental Hematology, National Clinical Research, Center for Hematological Disorders, Institute of Hematology and Blood Diseases Hospital, Chinese Academy of Medical Sciences and Peking Union Medical College, 300020 Tianjin, China; 4 Department of Hematology, Shandong Provincial Hospital Affiliated to Shandong First Medical University, 250021 Jinan, Shandong China; 5 Division of Hematology & Oncology, Department of Internal Medicine, Kaohsiung Medical University Hospital, Kaohsiung Medical University, Kaohsiung, 80708 Taiwan; 6 Faculty of Medicine, College of Medicine, Kaohsiung Medical University, Kaohsiung, 80708 Taiwan; 7 Novartis Institutes for Biomedical Research, 250 Massachusetts Avenue, Cambridge, MA 02139 USA

**Keywords:** Cancer immunotherapy, Immunotherapy

## Abstract

We here defined the impacts of γ-secretase inhibitors (GSIs) on T-cell-dependent BCMA-specific multiple myeloma (MM) cell lysis and immunomodulatory effects induced by bispecific antibodies (BisAbs). GSIs-induced membrane BCMA (mBCMA) accumulation reached near maximum within 4 h and sustained over 42h-study period on MM cell lines and patient MM cells. GSIs, i.e., 2 nM LY-411575 or 1 μM DAPT, robustly increased mBCMA densities on CD138^+^ but not CD3^+^ patient cells, concomitantly with minimum soluble/shed BCMA (sBCMA) in 1 day-culture supernatants. In ex vivo MM-T-cell co-cultures, GSIs overcame sBCMA-inhibited MM cell lysis and further enhanced autologous patient MM cell lysis induced by BCMAxCD3 BisAbs, accompanied by significantly enhanced cytolytic markers (CD107a, IFNγ, IL2, and TNFα) in patient T cells. In longer 7 day-co-cultures, LY-411575 minimally affected BCMAxCD3 BisAb (PL33)-induced transient expression of checkpoint (PD1, TIGIT, TIM3, LAG3) and co-stimulatory (41BB, CD28) proteins, as well as time-dependent increases in % effector memory/central memory subsets and CD8/CD4 ratios in patient T cells. Importantly, LY41157 rapidly cleared sBCMA from circulation of MM-bearing NSG mice reconstituted with human T cells and significantly enhanced anti-MM efficacy of PL33 with prolonged host survival. Taken together, these results further support ongoing combination BCMA-targeting immunotherapies with GSI clinical studies to improve patient outcome.

## Introduction

Since 2015, B-cell maturation antigen (BCMA), a very selective plasma cell (PC) receptor highly expressed in human multiple myeloma (MM) cells, has been the top target antigen for the novel monoclonal antibody (MoAb)- and cell-based immunotherapies [1–6]. The first BCMA-specific Ab drug conjugate (ADC) belantamab mafodotin was approved by FDA to treat heavily pretreated RRMM in 2020 [7], quickly followed by the approval of the anti-BCMA chimeric antigen receptor (CAR)-T therapies idecabtagene vicleucel (ide-cel) [8, 9] and ciltacabtagene autoleucel [10], as well as the breakthrough therapy designation of BCMA-targeting CD3-engaging bispecific Ab (BisAb) teclistamab [11] and trispecific Ab (TriTAC® HPN217) [12] most recently. These impressive results indicate that high specificity and cell surface density of BCMA protein are key determinants for the efficacious mono-immunotherapy for MM. Recent preclinical studies also demonstrate that BCMA-targeting agents trigger immunomodulatory effects to mitigate the immunosuppressive MM bone marrow (BM) microenvironment and prolonged animal survival when combined with current anti-MM drugs including bortezomib, immunomodulatory drugs (lenalidomide, pomalidomide), or CD38 MoAb daratumumab [13–16]. These studies further support combination BCMA-directed therapies with current stand-of-care anti-MM drugs to further enhance mono-immunotherapy efficacy.

However, membrane BCMA (mBCMA) receptor molecule is constantly cleaved by γ-secretase (GS), an intramembrane multi-subunit protease complex, and its extracellular portion with part of the transmembrane domain is shed to cell culture media or the circulation to form soluble BCMA (sBCMA) [17, 18]. The release of sBCMA may contribute to MM immunodeficiency by sequestering B-cell activating factor and a proliferation-inducing ligand [19]. Elevated sBCMA levels are found in serum samples of MM patients than healthy individuals [20] and further associated with myeloma burden and poorer survival [20–23]. These clinical results also indicate that sBCMA could serve as a trap to compete binding of BCMA-targeting drugs, thereby reducing their therapeutic promises. Moreover, decreased expression or loss of the antigen BCMA have been reported in patients relapsed from BCMA CAR T treatments [22, 24, 25].

We thus here defined the therapeutic potencies of small molecule GS inhibitors (GSIs) to increase mBCMA and counteract sBCMA inhibition in T-cell dependent cytotoxicity (TDCC) of BCMAxCD3 BisAbs against MM cell lines and patient MM cells in multiple preclinical human MM models in vitro and in vivo. A single GSI treatment rapidly enhanced mBCMA and eliminated sBCMA, leading to an improved BCMAxCD3 BisAb-induced MM cell lysis while sparing patient T-cell activation and differentiation in ex vivo co-cultures. GSI significantly augmented the in vivo anti-MM activity of a single sub-curative concentration BCMAxCD3 BisAb and prolonged host survival, further supporting ongoing combination clinical trials to improve patient outcome.

## Materials and methods

### Cell lines and primary cells

All MM cell lines (American Type Culture Collection and DSMZ German Collection) express various levels of BCMA [14, 15]. CRISPR/CAS9 technique was used to knock down BCMA expression to generate BCMA knock-down (KD) H929-KD [14] and U266-KD cells.

Samples from normal donors and MM patients were obtained after informed consent was provided, in accordance with the Declaration of Helsinki and under the auspices of a Dana-Farber Cancer Institute (DFCI) Institutional Review Board approved protocol.

Culture supernatants of MM cell lines and BMMCs of MM patients in 96-well culture plates (10^5^ cells per well) were collected for sBCMA measurement. Various concentrations of recombinant BCMA (sBCMA) (R&D Systems) were used to test its impact on MM cell killing by BCMAxCD3 vs. control BisAbs.

### Reagents and compounds

Nine different GSIs [26, 27] were tested for their potencies in blocking cleavage of mBCMA in MM cells. BCMAxCD3 BisAbs PL33 and ER26 were made with an anti-BCMA Fab (clone J6M0) [28] and an anti-CD3 scFv (clone SP34) [29, 30] on a human IgG backbone. They only differ in the pairing of Fc silencing mutations (DAPA vs. N297A) [31]. BQ76 and BU76 were made with anti-BCMA Fabs (clone 17A5 for BQ76 [32] and clone C11D5 for BU76 [33] with an anti-CD3 scFv (clone SP34) on a human IgG backbone.

### Flow cytometric (FC) data acquisition and analysis

Protein expression levels of mBCMA and mCD138, live vs. dead cell confirmation, redirected T-cell-dependent cytotoxicity (RTCC or TDCC) assays under mono- or combination treatment settings, autologous patient MM cell lysis, degranulation (CD107a mobilization) and intracellular Th1-cytokine expression of T effector cells, T-cell phenotype and memory cell differentiation, as well as CD8/CD4 ratios were evaluated by quantitative FC analysis.

The density of mBCMA molecule on MM cell surface was also quantitated by PE Phycoerythrin Fluorescence Quantitation Kit (BD Biosciences) and shown as Abs bound per cell (Ab binding capacity, ABC).

All data of FC-based investigations were collected by BD FACSCanto™ II and BD LSRFortessa™ flow cytometers, and then analyzed by FlowJo V8.6.6 (BD Life Sciences) and FACS DIVA software (BD Biosciences).

### In vivo adoptive T-cell transfer (AdT)-NSG mouse model of MM

Animal study procedures and protocols were performed under approval by the Novartis Institutes for BioMedical Research Institutional Animal Care and Use Committee and in compliance with the Guide for the Care and Use of Laboratory Animals. In brief, NSG mice (Jackson Labs Bar Harbor, ME) received adoptive transfer of human PBMCs (15 × 10^6^/mouse) (AdT) 5 days before KMS11-luc MM cells were implanted subcutaneously into the right flank (5 × 10^6^ cells/mouse) (d0). When the tumor volume reached 198 ± 47 mm^3^, mice were randomized into indicated groups (10 mice/group): controls (tumor only and Tumor + PBMC) or treatments receiving either LY-411575 (3 mg/kg), PL33 (1 or 3 mg/kg), or a combination of both. LY-411575 was administered for 2 consecutive days by oral gavage: 24 h before (d8) and the day when PL33 was administered (d9) as a single IV injection into the lateral tail vein 2 h post the second dose of LY-411575.

### Statistics

Unless otherwise specified, all data were analyzed and graphed using GraphPad Prism 9.0.1. *P*-value < 0.05 was considered statistically significant. Multiple groups (≥3) were analyzed by one-way analysis of variance (ANOVA), and paired groups were analyzed by two-way ANOVA or Student *t*-test.

Anti-tumor activity in vivo was evaluated using one-way analysis of variance with the Tukey post test, or an unpaired *t*-test with assumed similar variance. Kaplan–Meier Survival statistics were analyzed using Log-rank test with all pairwise multiple comparison ad hoc (Holm–Sidak method).

## Results

### GSIs significantly enhanced membrane BCMA (mBCMA) density and depleted shedding of BCMA (sBCMA) from MM cells

**Fig. 1:**
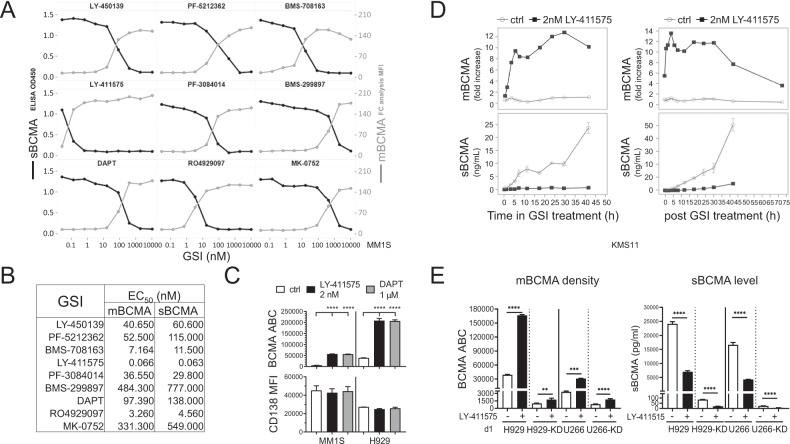
GSIs significantly enhanced mBCMA densities on MM cell lines and depleted sBCMA in paired cell culture supernatants. MM cell lines (**A**–**C**: MM1S; **D**: KMS11; **C**, **E**: H929; **E**: U266) were treated with various concentrations of the indicated GSI (**A**, **B**, 9 different GSIs (0.01–10,000 nM); **C**–**E** 2 nM LY-411575; **C** 1 μM DAPT) for 1 day (**A**–**C**, **E**) or indicated time periods (**D**) followed by flow cytometry (FC) analysis to determine geometric mean fluorescence intensity (MFI) of BCMA (mBCMA) (**A**, gray line, right Y axis; **D**, upper panels) and CD138 (**C**, lower panel). Fold-changes in MFI (to time 0) are shown in **D** (upper panels). Shed sBCMA concentrations in the paired cell culture supernatants were determined by BCMA-specific ELISA (**A**, black lines, left Y axis; **D**, lower panels; **E**, right). **B** EC_50_ (nM) values of these GSIs indicated that LY-411575, most potently, increased mBCMA and diminished sBCMA. Cell membrane BCMA molecules were further quantified by FC analysis and shown as BCMA antibody binding capacity (ABC) in **C** (upper panel) and **E** (left). **D** Shown are mBCMA MFI fold increases (upper panels) and measurements of sBCMA (lower panels) at indicated LY-411575 (black) treatment time periods (left panels) and indicated time periods (up to 75 h) after removal of LY-411575 (right). LY-411575 was pretreated for 1 day prior to washing (right). Data of vehicle control media (ctrl, gray) was also shown. **E** Paired control vector-transfected and BCMA-knockdown (KD) H929 and U266 MM cells were used. Three independent experiments were done with each treatment condition in triplicate. Data are presented as means ± standard deviations (SDs) (error bars). **P < 0.01; ***P < 0.001; ****P < 0.0001.

Using flow cytometry (FC) and ELISA analysis, all 9 GSIs (0.1–10,000 nM), in a dose-dependent manner, increased geometric mean fluorescence intensity (MFI) of BCMA on MM1S cell membrane and decreased sBCMA concentrations in culture supernatants, following 1 day treatment (Fig. 1A). ED_50_ values are comparable for mBCMA accumulation and sBCMA reduction, despite wide ranges of potencies (0.07−777 nM, Fig. 1B). LY-411575 is most effective, showing ~2-log greater potency than DAPT in all tested MM cell lines (Supplementary Fig. S1A–C).

In MM1S cells, further quantitation of mBCMA molecules as indicated by BCMA ABC showed that 2 nM LY-411575 significantly increased mBCMA molecules by ~4-fold (Supplementary Fig. S1C) after 4 h treatment and further enhanced by ~10-fold (Fig. 1C, Supplementary Fig. S1C, D) at d1 when sBCMA level was decreased by ~14-fold (Supplementary Fig. S1D). In H929 cells, 1 day treatment with GSI still enhanced mBCMA molecules by 4.7–5.3-fold while mCD138 levels were unchanged (Fig. 1C and Supplementary Fig. S1C).

The kinetics of GSI activity was next studied over a 42h-period in KMS11 cells cultured with 2 nM LY-411575 (Fig. 1D, left panels) or 200 nM PF03084014 (Nirogacestat) (Supplementary Fig. S1E). Only in the presence of GSI, mBCMA expression was enhanced over time (upper panel) whereas culture supernatant sBCMA levels stayed minimal (lower panel). Without GSIs, sBCMA concentrations steadily increased. GSI upregulated mBCMA expression as early as 1 h and near maximal level was reached at 4–6 h. GSIs continued to increase mBCMA with decreasing sBCMA levels for over 30 h. To determine how long the effect of GSI treatment persists, LY-411575 was washed out after 1 day treatment, and drug-free growth media was replenished (Fig. 1D, right panels). Membrane BCMA density on LY-411575-pretreated KMS11 cells persisted at ~12-fold higher levels than untreated cells till 30 h before decreasing. Meanwhile, mBCMA baseline levels in untreated cells remained constant over time. Concentrations of sBCMA remained low in LY-411575-pretreated MM cell culture supernatants while significantly increasing in untreated cell media.

We next examined the efficacy of GSI treatment in BCMA-knockdown (KD) H929-KD and U266-KD MM cells (~39- and 4-fold lower mBCMA, compared with their respective parental cells, Fig. 1E, left). LY-411575 treatment for 1 day still potently increased mBCMA densities on all these MM cells (*P* < 0.01) (Supplementary Fig. S1F). Levels of sBCMA in paired culture supernatants were all significantly diminished (*P* < 0.0001, Fig. 1E, right).

### The cytolytic activity of BCMAxCD3 bispecific antibodies (BisAbs) against MM cells was significantly enhanced by GSIs

**Fig. 2:**
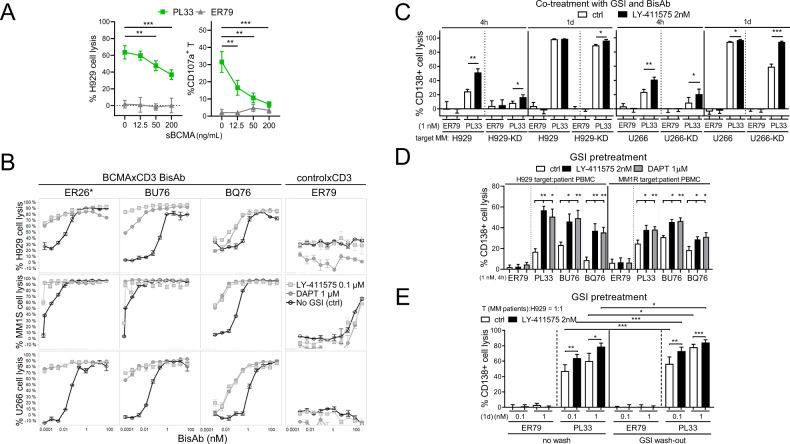
BCMAxCD3 bispecific antibodies (BisAbs)-induced MM cytolysis was significantly enhanced by GSI, in the presence of MM patient effector cells. **A** Serial dilutions of recombinant BCMA protein (sBCMA, 12.5–200 ng/mL) were added for 1 day in the co-cultures of patient T cells (*n* = 5) with H929 target MM cells (E:T = 1:1) in the presence of PL33 (BCMAxCD3, green square) vs. ER79 (non-targeting controlxCD3, gray triangle) BisAbs. Quantitative FC-based redirected T-cell cytotoxicity (RTCC) assays were used to determine % lysis of CD138^+^ target MM cells. Shown are summary data of % lysis of H929 cells (left) and CD107a degranulation on patient T cells (right). **B** Quantitative bioluminescence (BLI)-based RTCC assays were used to determine % lysis of 3 indicated MM target cells by T cells (*n* = 3) (E:T = 3:1) in the presence of 3 other BCMAxCD3 (ER26, BU76 or BQ76) clones vs. ER79, with or without 0.1 μM LY-411575 (gray square) or 1 μM DAPT (gray circle). No GSI control media (ctrl, black) was included. *ER26 has the same anti-BCMA Fab as PL33 used in all other figures. **C**–**E** Quantitative FC-based RTCC assays were used to determine % lysis of CD138^+^ target MM cells. **C** PL33 or ER79 (1 nM) were added for 4 h or 1 day in the co-culture of patient T cells (*n* = 5) with indicated paired MM target cells (control vector-transfected and BCMA-KD) (E:T = 3:1), in the presence of 2 nM LY-411575 or ctrl media. **D** H929 and MM1R target cells were pretreated with LY-411575 (2 nM) or DAPT (1 μM) followed by 4 h-co-incubation with MM patient PBMCs (*n* = 8) (E:T = 10:1) in the presence of indicated BisAbs (1 nM). (**E**) H929 target cells were pretreated with LY-411575 (2 nM) for 1 day. LY-411575 was then washed out (wash-out) or not (no wash) prior to 1 day-co-culture with MM patient T cells (*n* = 5) (E:T = 1:1) in the presence of PL33 vs. ER79. Multiple independent experiments using effector cells from MM patients (**A** *n* = 5; **C** *n* = 5; **D** *n* = 8; **E** *n* = 5) or normal donors (**B**, *n* = 3) were done with each treatment condition in triplicate. Data are presented as means ± SDs (error bars). *P < 0.05; **P < 0.01; ***P < 0.001; ****P < 0.0001; ns, not significant.

Using quantitative FC-based redirected T-cell cytotoxicity (RTCC) assays, we next determined effects of exogenous BCMA protein (sBCMA) on % loss of viable CD138^+^ target cells (% H929 cell lysis) by patient T cells (*n* = 5) (E:T = 1:1) in the presence of the BCMAxCD3 BisAb PL33 vs. non-targeting control ER79. The addition of sBCMA (0–200 ng/mL) significantly inhibited % CD138^+^ cell lysis (*P* < 0.01) and % CD107a degranulation in patient T cells (*P* < 0.006), starting from 12.5 ng/mL sBCMA (Fig. 2A).

Using bioluminescence (BLI)-based RTCC assays, PL33 specifically induced dose-dependent killing of luciferase (luc)-expressing MM cells by normal donor T cells (E:T = 1:1), whereas ER79 did not (data not shown). Co-treatments with 0.1 µM LY-411575 or 1 µM DAPT consistently augmented BCMAxCD3 BisAb-induced MM target cell lysis by T cells (Fig. 2B). EC_50_ values of all other tested BCMAxCD3 BisAb clones (ER26, BU76, and BQ76) were decreased by as much as 10-fold for GSI treatment vs. control. In contrast, ER79, either with or without GSIs, induced no MM-specific cytolysis.

We next showed that % CD138^+^ cell lysis induced by PL33 vs. ER79 was augmented as early as 4 h with co-treatments with 2 nM LY-411575 or 1 μ DAPT in co-cultures of 2 target MM cell lines with PBMCs (E:T = 6:1) or T cells (E:T = 3:1) from MM patients (*n* = 3) (Supplementary Fig. S2A). GSI alone induced neither MM cell lysis without BisAbs, nor MM cell apoptosis (*n* = 3) in the absence of T cells following 3 day-incubation (Supplementary Fig. S2B, C).

Under similar suboptimal test conditions, PL33 induced earlier and higher % lysis of H929 vs. U266 target cells, associated with increased mBCMA levels (Fig. 2C). When H929-KD or U266-KD target cells were co-cultured with patient T cells (*n* = 5) (E:T = 1:1), co-treatments with LY-411575 still significantly enhanced PL33-induced MM cell killing as early as 4 h (*P* < 0.03) and continued to increase at 1 day (*P* < 0.05), reaching maximal lysis associated with increased % CD107a^-^ patient T cells (Supplementary Fig. S2D). GSI-pretreated MM target cells again significantly augmented BCMA-specific MM cell lysis induced by patient T cells (*P* < 0.05), regardless which BCMAxCD3 BisAb or target MM cells were used (Fig. 2D and Supplementary Fig. S2E).

H929 cells pretreated with LY-411575 were also washed with or without fresh culture media before co-incubation with patient T cells (E:T = 1:1), in the presence of PL33 vs. ER79. GSI effect was sustained in PL33-induced H929 cell lysis (*P* < 0.04) (Fig. 2E) and CD107a surface expression on patient T cells (Supplementary Fig. S2F, *P* < 0.05) at 1 day post GSI wash-out. Similar GSI-increased effects were seen when T cells from normal donors were used (Supplementary Fig. S2G).

### GSI treatment specifically enhanced mBCMA accumulation on patient MM cells and depleted sBCMA

**Fig. 3:**
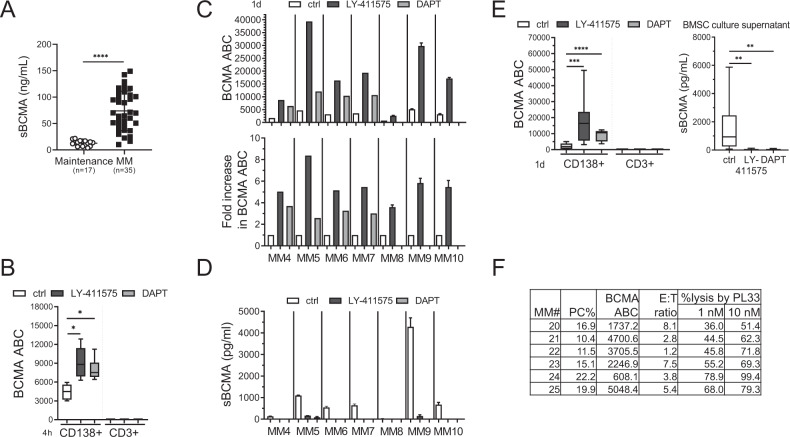
GSIs robustly upregulated mBCMA densities on patient MM but not T cells, significantly associated with sBCMA elimination in supernatants of 1 day-patient cell cultures. **A** Serum sBCMA (ng/mL) levels were determined in patients with active MM diseases (*n* = 35) and at maintenance after treatment response (*n* = 17). **B**–**E** BMMCs (10^5^ cells per well in 96-well culture plates) from MM patients were treated with LY-411575 (2 nM) or DAPT (1 µM) for 4 h (**B**, *n* = 3) or 1 day (**C**, **D** *n* = 7, **E**, *n* = 9) followed by quantitative FC analysis (**B**, **C**; **E**, left panel) and ELISA (**D**, **E**, right panel) to determine BCMA ABC on CD138^+^ (**B**, **C**; **E**, left panel) vs. CD138^−^CD3^+^ T-cell subsets (**B**; **E**, left panel) and sBCMA concentrations in 1 day-culture supernatants (**D**; **E**, right panel), respectively. **F** PL33 of 1 and 10 nM was added in BMMCs collected from more MM patients for 1 day followed by quantitative FC analysis to determine % autologous CD138^+^ cell lysis. Shown are also % PC (plasma cell, CD138^+^), BCMA ABC, and CD3^+^ T cells in these samples (MM20-25, *n* = 6). Multiple experiments were done in triplicate at each treatment using multiple patient materials. Data are presented as means ± SDs (error bars). *P < 0.05; **P < 0.01; ***P < 0.001; ****P < 0.0001.

MM patient serum sBCMA levels (Fig. 3A) were significantly elevated in cohorts with active disease (*n* = 35, ranged from 10.25 to 150.2 ng/mL) vs. maintenance therapy (*n* = 17, ranged from 3.69 to 21.69 ng/mL) (*P* < 0.0001, 73.85 ± 37.34 vs. 12.79 ± 5.24 ng/mL), indicating that sBCMA levels correlate with patient myeloma burden. Moreover, LY-411575 or DAPT treatment for 4 h in BMMCs from MM patients rapidly increased BCMA ABC values on CD138^+^ patient cells, from 4443 ± 1206 to 9137 ± 2581 (*P* < 0.03) and to 8002 ± 1747 (*P* < 0.03), respectively (Fig. 3B). No BCMA was detected on CD138-CD3^+^ patient T cells, with or without GSI treatment.

GSI treatment for 1 day was next done in additional MM BMMCs (*n* = 7) with baseline BCMA ABC ranging from 714 to 5311 on CD138^+^ cell membrane (Fig. 3C, upper panel). LY-411575 (MM4-10) and DAPT (MM4-7, *n* = 4) upregulated mBCMA levels on patient MM cells by ~3.9–8.4- and 2.6-3.7-fold, respectively (Fig. 3C, lower panel). Furthermore, concentrations of sBCMA negatively associated with mBCMA levels, being undetectable in ~75% culture supernatants treated with GSI (Fig. 3D).

No mBCMA was detected on CD3^+^ T cells from another 9 MM patients with baseline mBCMA molecules on the paired CD138^+^ cells ranging from 414 to 5011 (Fig. 3E, left panel). Following GSI treatments for 1 day, mBCMA levels on CD138^+^ cells were increased from 3034 ± 1694 to 18190 ± 12085 for LY-411575 (*P* < 0.002) and to 10114 ± 2437 for DAPT (*P* < 0.0001). GSIs specifically upregulated mBCMA expression on CD138^+^ cells by as much as 22-fold, without impacting paired patient T cells. Meanwhile, sBCMA levels in matched 1 day-culture supernatants of patient BMMCs were decreased from ~6000 pg/mL (mean: 1597 pg/mL; SD: 1755 pg/mL) to undetectable in 7 out of 9 samples for LY-411575 (mean: 30.81 pg/mL; SD: 46.75 pg/mL) or DAPT treatment (20.96 pg/mL; SD: 33.58 pg/mL) (*P* < 0.02, Fig. 3E, right panel). Thus, GSIs effectively blocked BCMA shedding from patient MM cells.

Following 1 day-incubation, PL33-induced patient autologous lysis were next shown using BMMCs from 6 RR MM patients with BCMA ABC ranging from 608 to 5048 on CD138^+^ PCs (Fig. 3F). As expected, higher PL33 concentrations triggered higher autologous patient cell lysis.

### GSI upregulated autologous patient MM cell lysis induced by BCMAxCD3 BisAb

**Fig. 4:**
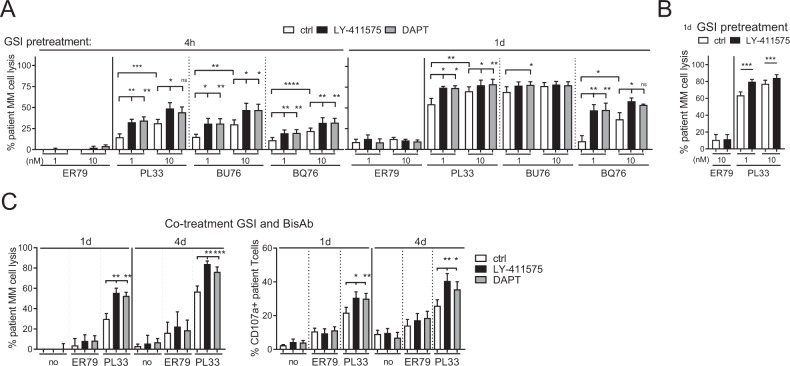
Pretreatment or co-treatment with GSIs further augmented autologous patient MM cell lysis induced by BCMAxCD3 BisAbs. BMMCs of MM patients (**A**, *n* = 6; **B**, *n* = 5; **C**, *n* = 8) were pretreated (**A**, 4 h and 1 day; **B**, 1 day) or co-treated (**C**) with GSIs (2 nM LY-411575 or 1 µM DAPT). Indicated BisAbs (1, 10 nM in **A**, **B**; 1 nM in **C**) were added with PBMCs from the paired individuals for 1 day (**A**, **C**) or 4 days (**C**). Quantitative FC analysis was used to determine % patient MM cell (CD138^+^) depletion (**A**, **B**; **C**, left panel) and CD107a degranulation in autologous CD3^+^ patient T cells (**C**, right panel). Experiments were done in triplicate for each condition using multiple patient samples. Data are presented as means ± SDs (error bars). *P < 0.05; **P < 0.01; ***P < 0.001; ****P < 0.0001; ns, not significant.

MM BMMCs (*n* = 6) were next pretreated with LY-411575 or DAPT for 4 h and 1 day prior to the addition of three indicated BCMAxCD3 BisAbs vs. ER79 with matched patient PBMCs. These three BCMAxCD3 BisAbs induced autologous CD138^+^ patient cell lysis, with higher potency of PL33 and BU76 compared with BQ76 (Fig. 4A). GSI significantly enhanced autologous patient MM cell lysis induced by BCMAxCD3 BisAbs as early as 4 h and continued at 1 day. In contrast, pretreatments with GSI did not further change % background lysis. In five more MM patient BMMCs, pretreatment with LY-411575 for 1 day also enhanced % autologous patient cell lysis by PL33 vs. ER79 (Fig. 4B). We next co-treated BMMCs from RRMM patients (*n* = 8) with LY-411575 or DAPT during PL33 (1 nM)-induced RTCC assays for 1 day and 4 day (Fig. 4C, left). Co-treatment with GSI still significantly augmented PL33-induced autologous patient MM cell lysis at d1 (*P* < 0. 004) and d4 (*P* < 0.003). This was associated with increased % CD107^+^ patient T cells at d1 (*P* < 0.015) and d4 (*P* < 0.004) (Fig. 4C, right).

### LY-411575 co-treatment minimally affected T-cell checkpoint markers and T-cell differentiation in the MM-PBMC/T co-cultures

In 1 day-co-cultures of MM1S-PBMCs (*n* = 3, E:*T* = 5:1), PL33, but not ER79, enhanced expression of checkpoint markers (PD1, TIGIT, LAG3) in T cells, which was slightly decreased in the presence of LY-411575 (Supplementary Fig. S3). Also, PL33 induced % effector memory (EM, CD45RA^−^CD62L^−^) and/or terminal effector memory RA (TEMRA, CD45RA^+^CD62L^−^) from naïve (CD45RA^+^CD62L^+^) subsets in these activated T cells of normal donor and patient PBMCs. LY-411575 further enhanced % EM and/or TEMRA T-cell subsets induced by PL33, correlating with further MM cell lysis.

**Fig. 5:**
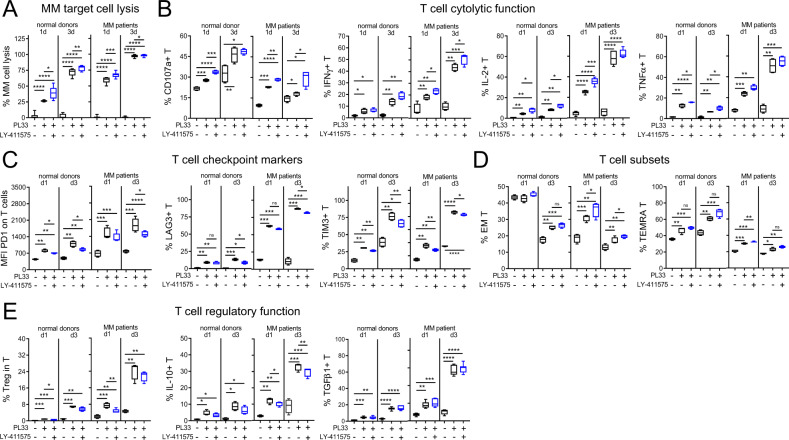
LY-411575 co-treatment upregulated PL33-induced cytolytic markers without further increased expression of immune checkpoint molecules and regulatory cell subsets in T cells in 3 day-co-cultures. MM1S target cells were co-cultured with T cells from normal donors (*n* = 5) or MM patients (*n* = 5) (E:T = 1:1) for 1 and 3 days, in the presence (+) or absence (−) of 1 nM PL33 with or without 2 nM LY-411575. Shown are summary data (means ± SDs) for % CD138^+^ target cell lysis (**A**), 4 key markers for cytolytic function of T cells (**B**), 3 major checkpoint molecules on T cells (**C**), % differentiated T-cell subsets including effector memory (EM, CD45RA^−^CD62L^−^) and terminal effector memory RA (TEMRA, CD45RA^+^CD62L^−^) (**D**), as well as main molecules (IL-10, TGFβ) related to regulatory T (Treg, CD4^+^CD25^+^FoxP3^+^) cell function (**E**). Five independent experiments using T cells from normal donors (left) and MM patients (right) were done with each condition in triplicate. *P < 0.05; **P < 0.01; ***P < 0.001; ****P < 0.0001; ns, not significant.

MM1S target cells were further co-cultured with T cells from five additional individuals of normal donors or MM patients, for 1 and 3 days at lower E:T ratio of 1:1 and PL33 concentration, with or without LY-411575. PL33-induced MM1S cell lysis was associated with enhanced CD107a degranulation and Th1-type cytokine (IFNγ, IL-2, TNFα) production in T cells from both sources (Fig. 5A, B). Importantly, PL33-induced MM1S cell killing by patient T cells was further enhanced with LY-411575 co-treatment at d1 and continued at d3, associated with further increased cytolytic markers. Expression of PD1, LAG3, and TIM3, continued to increase in PL33-activated T cells from d1 to d3 (Fig. 5C, *P* < 0.01). LY-411575 did not reduce % of EM or TEMRA subsets induced by PL33 (Fig. 5D). Moreover, PL33 induced regulatory T (Treg) cell subsets from d1 and continued at d3, while LY-411575 co-treatment did not further increase % Treg, IL-10^+^, or TGFβ^+^ T-cell subsets (Fig. 5E).

**Fig. 6:**
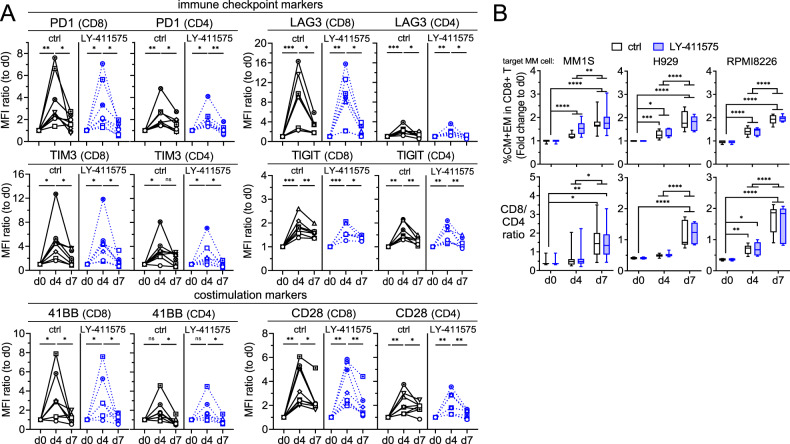
LY-411575 minimally affected PL33-induced transient expression of checkpoint and co-stimulatory markers, as well as time-dependent increases in memory cell differentiation and CD8/CD4 ratios in patient T cells in longer 7 day-co-cultures. **A** Mixtures of patient T cells (*n* = 8) with target MM1S cells (E:T = 1:1) were treated with 1 nM PL33 in control (ctrl, black) or LY-411575 (2 nM, blue)-containing media on day 0 (d0). In 7 day time course expression studies, quantitative FC analysis was used to determine serial changes of immune checkpoint markers (PD1, TIM3, LAG3, TIGIT) and co-stimulatory markers (4-1BB, CD28) on CD8 (left in each marker panel) and CD4 (right in each marker panel) T cells. Shown is the relative expression of each marker by MFI ratios, which was normalized to the MFI values of ctrl groups on d0. The serial changes of each marker were analyzed by Wilcoxon matched pairs signed rank test. Different symbols represent T cells from different individuals. **B** Indicated MM target cells (*n* = 3) were co-cultured with patient T cells for 7 day (*n* = 8–11) in the presence of 1 nM PL33, in ctrl or LY-411575-containing media. Shown are % central memory (CM, CD45RA^−^CD62L^+^) and % EM (CD45RA^−^CD62L^−^), as well as CD8/CD4 ratios. Eight to 11 independent experiments were done in triplicates at each time point. *P < 0.05; **P < 0.01; ***P < 0.001; ****P < 0.0001; ns, not significant.

Further 7 day-co-cultures were done to study the serial changes in expression of key immune checkpoints (PD1, LAG3, TIM3, TIGIT) and co-stimulatory proteins (41BB, CD28) using patient T cells (*n* = 8) and MM1S cells (E:T = 1:1) treated with PL33, in the presence or absence of LY-411575. PL33 induced transient expression of these immune activation and regulatory proteins (Fig. 6A). Higher induction of these proteins was seen in PL33-activated CD8 vs. paired CD4 patient T cells. LY-411575 did not alter transient expression patterns of these markers induced by PL33 in patient T cells.

In co-culture of MM1S cells with T cells, PL33 significantly increased % T cells with central memory characteristics (CM, 45RA^−^CD62L^+^) at d7 following differentiation into EM and TEMRA T-cell subsets at d1 (Supplementary Fig. S4). We further examined impacts of LY-411575 on PL33-increased % CM + EM CD8 T subsets as well as CD8/CD4 ratios in 7 day-co-cultures of patient T cells (*n* = 8–11) with 3 target MM cells. PL33 significantly increased % CM + EM CD8 patient T subsets at d4, with continued increase to d7 (Fig. 6B, upper panels). During this period, CD8/CD4 ratios also continued to increase (*P* < 0.01 at d7) (Fig. 6B, lower panels). LY-411575 co-treatment impacted neither % CM + EM CD8 patient T cells nor CD8/CD4 ratios induced by PL33.

### GSI quickly depleted sBCMA and enhanced the anti-MM efficacy of BCMAxCD3 BisAb in vivo with extended host survival

**Fig. 7:**
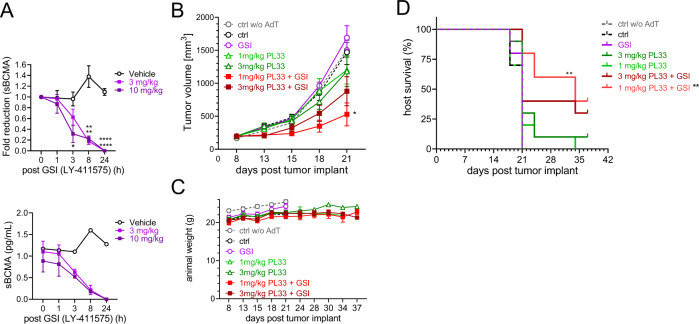
GSI rapidly and effectively cleared sBCMA released from MM tumors in mouse serum and further improved in vivo anti-MM activity of BCMAxCD3 BisAb with extended host survival. **A** NSG mice were inoculated with KMS11-luc cells subcutaneously on the right flank at d0 and administered a single dose of GSI (3 (light purple square) and 10 (dark purple square) mg/kg LY-411575), or vehicle treated control (black open circle) when tumor volume reached ~325 mm^3^. Serum was serially collected following indicated time periods to evaluate sBCMA levels. Shown are fold reduction in sBCMA from baseline (time 0) (upper panel) and sBCMA pg/mL (lower panel). Data are presented as means ± SEMs (error bars). **B**–**D** NSG mice were injected with PBMCs (AdT) 5 days prior to KMS11-luc cell implantation on d0 and mice were randomized into their respective groups on d8 when average tumor size was ~200 mm^3^. LY-411575 (3 mg/kg, QDx2) was administered PO as a single agent GSI and in combination groups. On the following day (d9), the second dose of LY41175 was administered followed by a single dose of PL33 to the single agent PL33 groups and combination groups at a dose 1 or 3 mg/kg IV. Cohorts included: 3 mg/kg LY-411575 (purple circle), 3.0 mg/kg (dark green triangle) or 1.0 mg/kg (light green triangle) PL33, LY-411575 + 3 mg/kg PL33 (maroon solid square) LY-411575 + 1 mg/kg PL33 (red solid square), tumor only (ctrl, black circle), tumor without PBMCs (ctrl w/o AdT, gray circle). **B** Shown are mean tumor volumes (mm^3^) ± SDs (error bars) at following days vs. start of treatment. *P < 0.05 for tumor growth inhibition of single agent and combination treatments (one-way ANOVA followed by Tukey’s multiple comparison test and unpaired *t*-test between single agent PL33 compared to combination. **C** Weights of mice were followed. **D** Conditional survival plot with time to endpoint set when tumor burden (TB) reached ~1200 mm^3^. Kaplan–Meier and log-rank (Mantal–Cox) analysis followed by multiple comparison tests (Holm–Sidak method) was used to estimate median overall survival of animals (1 mg/kg PL33 with GSI, 34 days; the other 6 groups, 21 days) (*P* < 0.01). *P < 0.05, **P < 0.01, ***P < 0.001, ****P < 0.0001.

The in vivo kinetics of sBCMA depletion of GSI (LY-411575) was next evaluated at 3 and 10 mg/kg in a single dose pharmacodynamics (PD) study in NSG mice bearing KMS11-luc tumors (~325 mm^3^). Both LY-411575 concentrations were well tolerated in mice and induced >70% sBCMA clearance by 8 h after drug treatment, reaching a complete reduction through 24 h (*P* < 0.0001, Fig. 7A).

In vivo efficacy of combination BCMAxCD3 BisAb (PL33) at sub-curative concentrations (1 and 3 mg/kg) with GSI (LY-411575, 3 mg/kg) was subsequently determined in the KMS11-luc MM xenograft adoptive transfer (AdT) model. Mice reconstituted with human PBMCs were implanted with KMS11-luc MM cells and randomized into seven indicated groups (*n* = 10 mice per group) when tumors reached an average of ~200 mm^3^. LY-411575 was administered 1 day before and on the same day as a single PL33 treatment. All groups received human PBMCs (AdT) except tumor only (ctrl w/o AdT) group. Both untreated control cohorts (ctrl w/o AdT and ctrl groups) showed similar growth kinetics, indicating no allogeneic response (Fig. 7B). There was no anti-MM activity with single agent LY-411575, as tumor progression was comparable to two untreated controls. The mice receiving a single administration of PL33 at sub-curative concentrations also had minimal anti-tumor effects (% Treatment/Control (T/C) of 77.02 and 78.46, respectively, at d21), which were not statistically significant from two untreated controls or the single agent LY-411575.

Importantly, the 1 and 3 mg/kg PL33 in combination with LY-411575 did not show toxicity in animals (Fig. 7C) and further decreased tumor burden (%T/C 25.63 and 52.80, respectively, at d21) when compared with either agent alone, with the 1 mg/kg PL33 in combination showing stronger response compared to the 3 mg/kg PL33 in combination. The MM growth inhibition in the 1 mg/kg PL33 with LY-411575 showed statistically significant differences from the single agent LY-411575 and PL33 matched doses, as well as the untreated controls (at d21, *P* = 0.0.0012 for 1 mg/kg combination vs. GSI; *P* < 0.03 for 1 mg/kg combination vs. ctrl; *P* < 0.008 for 1 mg/kg combination vs. ctrl w/o AdT). Cohorts for two untreated controls and the LY-411575 single agent reached end tumor volume by d21, but the remaining groups were continued on the study to assess survival advantage (Fig. 7D). The combined 1 mg/kg PL33 with LY-411575 treatment significantly improved animal survival compared to 1 m/kg PL33 alone (*P* < 0.01). Additionally, at the time that all mice treated with 1 mg/kg PL33 alone (median survival day 21; 1PR + 0CR) had been removed from study due to tumor progression, 50% of animals in the group receiving combination 1 mg/kg PL33 with LY-411575 were still alive (median survival 34 days, 1PR + 4CR). The 3 mg/kg PL33 only and combination with LY-411575 resulted in 3PRs + 1CR and 5PRs + 2CRs, respectively, suggesting an improved treatment effect in the combination, even though statistical significance in host survivals was not realized. The Kapan–Meier Time to Endpoint plot indicated a trend toward prolonged survival in the combination groups over the single agent PL33.

## Discussion

We here show that GSIs effectively restored BCMA loss on the MM cell membrane by blocking GS cleavage in releasing sBCMA into culture supernatants of patient MM BMMCs. This potent GSI action led to enhanced anti-MM activity of BCMAxCD3 BisAb, without adverse impact on T-cell activation and memory cell differentiation in ex vivo 7 day-co-cultures. Importantly, a single low-dose GSI rapidly cleared sBCMA in serum samples of NSG mice bearing KMS11-luc myeloma. While two consecutive administrations of LY-411575 did not impact tumor growth, combination LY-411575 with a single sub-curative PL33 treatment significantly increased anti-MM efficacy of PL33 monotherapy, evidenced by extended host survival in the KMS11-luc MM xenograft-AdT mouse model.

The use of GSIs that were originally developed for Alzheimer’s disease and cancer is primarily based on the premise that GSIs act by inhibiting the cleavage of GS on Notch ligands, thereby resulting in Notch 1 signaling blockade [34–38]. However, their success in preclinical models has not yet translated into clinical benefit [39]. Despite disappointing clinical performance, our data here further support repurposing GSI use to effectively augment BCMA targeting to achieve more complete and durable MM cell lysis by BCMAxCD3 BisAbs, as recently demonstrated in BCMA CAR T therapies [40].

Because of its highest potency to block BCMA cleavage without direct apoptotic effects on MM cells and T cells, 2 nM LY-411575 (ED_50_ of 0.07 nM) was used in our studies. LY-411575 most efficiently retained mBCMA while diminished sBCMA for ~2 days. Accumulation of mBCMA molecules by LY-411575 quickly reached near maximum within 4 h and sustained with barely detectable sBCMA for over 42 h. Importantly, LY-411575 depleted sBCMA by >20–50-fold in patient samples, further confirming that GSI blocks BCMA shedding from MM cells in patients. Despite different kinetics seen in MM cells expressing various baseline BCMA levels or % BCMA^+^ patient CD138^+^ cells, GSIs robustly upregulated mBCMA densities and downregulated sBCMA in all MM cell lines and patient MM cells, regardless of disease and drug resistance statuses. As expected, other MM antigens including CD138, SLAMF7, CD38, and GPRC5D (data not shown) were unchanged since they were not the substrates for GS.

We further confirmed that mBCMA density is correlated with sBCMA concentration due to continuous GS cleavage under normal physiological conditions. Serum sBCMA levels were significantly associated with MM disease burden, supporting sBCMA as a valuable biomarker and highlighting sBCMA as a potential drug sink to abrogate effective BCMA-directed therapies. Indeed, sBCMA at patho-physiological concentrations (12.5–200 ng/mL) observed in MM patients inhibited MM cell lysis by BCMAxCD3 PL33 in a dose-dependent manner. Since RRMM patients have higher disease burden secreting even more sBCMA into the circulation [20, 21, 23], coupled with impaired effector T-cells, GSI treatment could provide a very promising strategy to overcome sBCMA interference, thereby allowing even efficient MM cell targeting and T effector cell engagement to kill MM cells by BCMAxCD3 BisAbs. Importantly, we showed that GSI potently led to increased autologous patient MM cell killing.

In 3 day-co-cultures, GSI did not further extend PL33-induced T-cell activation and checkpoint marker expression, suggesting that GSI minimally induced T-cell exhaustion. GSI further increased % EM and/or TEMRA T-cell subsets, associated with enhanced MM cell lysis. Consistent with our previous report on another BCMAxCD3 AMG 701 [14], PL33 also induced higher % of Treg, IL10^+^, and TGFβ^+^ T-cells at later time points. Deceased % T-cell subsets with inhibitory characteristics were seen in GSI treatment vs. control media cohorts. These findings are consistent with studies reporting that GSI (i.e., LY 411575, DAPT) treatment blocked expression of TGFβ1-induced Foxp3 and its downstream genes, as well as Treg suppression on naive T-cell proliferation [41, 42]. Moreover, LY-411575 did not alter transient induction patterns of key co-stimulatory and immune regulatory markers on patient CD8^+^ and CD4^+^ T-cells by PL33 in 7 day-co-cultures. It affected neither PL33-induced time-dependent increase in % CM + EM nor CD8/CD4 ratios in patient T cells at d7. Thus, GSI treatment spared BCMAxCD3 BisAb-induced patient effector T-cell function and memory cell differentiation, suggesting that combination therapy can trigger persistent anti-MM activity in the clinic.

Since inhibition of BCMA cleavage by GS is reversible, we performed two GSI doses in consecutive days to prevent tumor cells still escaping BCMAxCD3 BisAb recognition in vivo. The first dose shifted the dynamic ratio of sBCMA to mBCMA to enhance target antigen on the membrane and simultaneously decrease the sBCMA sink. The administration of the second dose of GSI ensured that this shift is maintained through the BisAb dosing interval. Thus, LY-411575 was administered once one day prior to PL33 treatment to decrease sBCMA sink and the following day to maintain sBCMA suppression. Importantly, compare with PL33 monotherapy, combination LY-411575 with a single sub-curative PL33 treatment significantly enhanced KMS11 MM cell growth and prolonged host survival in the AdT-NSG mouse model.

In conclusion, GSI prevents mBCMA loss from MM cells and overcomes the sBCMA decoy neutralization, thereby augmenting MM cell targeting and efficacy of BCMAxCD3 BisAbs. A single treatment with GSI at ~2-log lower drug concentration than used to induce anti-cancer activity maximizes mBCMA density without harming normal cells. This could avoid severe gastrointestinal toxicity of GSIs seen in earlier studies, which limits its potential clinical use [43–46]. Rationally incorporating GSI into all BCMA-targeting immunotherapy therefore represents a promising novel combination approach to further improve response durability and patient outcome in MM.

## Supplementary information

Supplement file

## Data Availability

The datasets generated during and/or analyzed during the current study are available from the corresponding author on reasonable request.
