# Supplementary material for: γ-secretase inhibitors augment efficacy of BCMA-targeting bispecific antibodies against multiple myeloma cells without impairing T-cell activation and differentiation
Source: Blood Cancer J. 2022 Aug 16;12(8):118. doi: 10.1038/s41408-022-00716-3 (PMC9381512; doi:10.1038/s41408-022-00716-3)
Supplement: Supplementary file 1 — Supplement file [file 41408_2022_716_MOESM1_ESM.docx]

**γ-secretase inhibitors augment efficacy of BCMA-targeting bispecific antibodies against multiple myeloma cells without impairing T-cell activation and differentiation**

Hailin Chen^1,2^, Tengteng Yu^1,3^, Liang Lin,^1^ Lijie Xing^4^, Shih-Feng Cho^5,6^, Kenneth Wen^1^, Kimberly Aardalen^7^, Adwait Oka^7^, Joni Lam^7^, Mike Daley^7^, Haihui Lu^7^, Nikhil Munshi^1^, Kenneth C Anderson^1^*, Yu-Tzu Tai^1^*

^1^Jerome Lipper Multiple Myeloma Center, LeBow Institute for Myeloma Therapeutics, Dana-Farber Cancer Institute, Harvard Medical School, Boston MA, USA

^2^Hematology Department, Yueyang Hospital of Integrated Traditional Chinese and Western Medicine, Shanghai University of Traditional Chinese Medicine, Shanghai, China.

^3^State Key Laboratory of Experimental Hematology, National Clinical Research, Center for Hematological Disorders, Institute of Hematology and Blood Diseases Hospital, Chinese Academy of Medical Sciences and Peking Union Medical College, Tianjin 300020, China

^4^Department of Hematology, Shandong Provincial Hospital Affiliated to Shandong First Medical, University, Jinan, 250021, Shandong, China.

^5^Division of Hematology & Oncology, Department of Internal Medicine, Kaohsiung Medical University Hospital, Kaohsiung Medical university, Kaohsiung 80708, Taiwan

^6^Faculty of Medicine, College of Medicine, Kaohsiung Medical University, Kaohsiung 80708, Taiwan

^7^Novartis Institutes for Biomedical Research, 250 Massachusetts Ave, Cambridge, MA02139, USA

*To whom correspondence should be addressed: Yu-Tzu Tai, PhD, Department of Medical Oncology, Dana-Farber Cancer Institute, M551, 450 Brookline Avenue, Boston, MA 02215. Phone: 617-632-3875; E-mail: yu-tzu_tai@dfci.harvard.edu; Kenneth C Anderson, M.D., Department of Medical Oncology, Dana-Farber Cancer Institute, M557, 450 Brookline Avenue, Boston, MA 02215 Phone: 617-632-2144; Email: Kenneth_anderson@dfci.harvard.edu

**Supplementary methods**

**Cell lines and primary cells**

MM cell lines^1-3^ were obtained from American Type Culture Collection (ATCC) and the DSMZ German Collection (Leibniz Institute DSMZ). They were grown in RPMI-1640 (Invitrogen, Carlsbad, CA) with 10% fetal bovine serum (Hyclone, Logan, UT), 2 mM/L L-glutamine, 100 U/ml penicillin and 100 µg/ml streptomycin (Invitrogen). They were routinely checked by Human Short Tandem Repeat (STR) DNA Profiling for their authenticity, as well as for mycoplasma contamination by PCR and the MycoAlert™ PLUS Mycoplasma Detection Kit from Lonza (Walkersville, MD).

Using density gradient centrifugation with Ficoll-Hypaque (GE Healthcare), mononuclear cells (MC) were isolated from peripheral blood (PB) and bone marrow (BM) collected in heparinized tubes. T cells (> 94% CD3^+^) from PBMCs of normal donors and MM patients were enriched using Human T Cell Enrichment Cocktail (RosetteSep™, STEMCELL) and used as effector cells in MM-T cell co-cultures.

**GSI treatment of KMS11 MM cells**

For GSI-dose response curves, KMS11 cells were cultured in 96-well plates at 50,000 cells per well in a final volume of 100 µL that included a 12-point, 5-fold serial dilution of GSIs in RPMI1640 supplemented with 20% FBS (Gibco #11875-085, Seradigm #1500-500). Top starting concentration of LY411575 (Sigma #SML0506) was 1 µM. Cells were incubated for 20h at 37°C/5% CO2. Cells were pelleted, supernatant collected for ELISA determination of shed BCMA (sBCMA) levels, and cell pellets stained for flow cytometry evaluation of BCMA membrane (mBCMA) expression levels.

For the time course experiments, KMS11 cells were cultured in a 6 well plate at 4 x 10^6^ cells/well in 4 mL of RPMI1640 supplemented with 20% FBS (Gibco #11875-085, Seradigm #1500-500) per well, and GSI was added when indicated. LY-411575 stock solution was prepared in DMSO at 10 mM and added to the cells at final concentrations of 2 nM. Cells were incubated at 37°C/5% CO_2_, and samples were collected at the following time points: 0, 1, 2, 4, 6, 8, 12, 18, 24, 30 and 42 hours. KMS11 cells were pre-treated overnight with LY411575 as described above. Cells were then washed twice and re-plated with fresh growth medium without GSI to the original 4 mL volume. Cells were incubated at 37°C/5% CO2, and samples were collected at the following time points: 0, 1, 2, 4, 6, 8, 12, 18, 24, 30 and 42 hrs. (0h is at the starting point after the overnight treatment and washing).

**Analysis of mBCMA *in vitro* by Flow cytometry (FC) analysis**

Cells were pelleted by centrifugation, and supernatants were transferred to a fresh plate and frozen at -20°C for later analysis by ELISA. Cell pellets were resuspended in 50 µL MACS buffer containing BSA (Miltenyi #130-091-222, 130-091-376) and stained with anti-BCMA-PE (Biolegend #357504, 3:50 dilution) for 30 minutes at 4°C. Cells were washed, fixed for 20 minutes in 10% neutral buffered formalin (VWR #16004-126), and stored at 4°C until all time points were collected. Samples from all time points were analyzed together by FC analysis on a BD LSR Fortessa instrument. FlowJo v10 software was used for analysis. The ratio of mean fluorescent intensity (MFI) of PE (BCMA) for GSI-treated wells was divided by the MFI for untreated KMS11 wells. These ratios were plotted in Tibco Spotfire or GraphPad Prism against the concentration of GSI.

Monoclonal antibodies (MoAbs) for analysis purchased from Biolegend (San Diego, CA) are listed below, and Abs against CD3, FOXP3, TGF-β1 were obtained from BD Biosciences.

| Target | Clone | Catalog number |
| --- | --- | --- |
| BCMA | 19F2 | 357504 |
| CD138 | MI15 | 356508 |
| CD4 | RPA-T4 | 300532 |
| CD8 | HIT8a | 300912 |
| CD62L | DREG-56 | 304806 |
| CD45RA | HI100 | 304126 |
| CD107a | H4A3 | 328620 |
| IFNγ | 4S.B3 | 502506 |
| TNFα | MAb11 | 502909 |
| PD1 | EH12.2H7 | 329908 |
| TIM3 | F38-2E2 | 345012 |
| LAG3 | 11C3C65 | 369306 |
| CD28 | CD28.2 | 302926 |
| 4-1BB | 4B4-1 | 309810 |
| IL-10 | JES3-9D7 | 501420 |
| Annexin V |  | 640908 |
| CD3 | SK7 | 641397 |
| FOXP3 | 259D/C7 | 560045 |
| TGF-β1 | TW4-9E7 | 562962 |

Annexin V (Biolegend) and LIVE/DEAD™ Fixable Aqua Dead Cell Stain Kits (Thermo Fisher Scientific) were routinely used to distinguish apoptotic from viable cells.

**Measurement of sBCMA levels by ELISA**

Levels of sBCMA in supernatants collected and frozen at the various time points were determined by ELISA following vendor supplied protocol (R&D Systems #DY193). Briefly, recombinant human BCMA-Fc protein was included in the kit and used to generate a standard curve. Collected samples were assayed and sBCMA concentrations extrapolated from the standard curve. Quantified values as determined by the kit were divided by 5.5 to correct for a molecular mass difference between BCMA-Fc fusion protein used in the kit as a standard curve (32,554.6 Da) and the mass of endogenously shed BCMA extra-cellular domain (5,899.3 Da). The results were plotted in Tibco Spotfire or GraphPad Prism against the concentration of GSI.

**BLI-based RTCC/TDCC assay**

Target human MM cell lines expressing various levels of human BCMA (NCI-H929, MM1S, and U266B1) were all transduced to constitutively express luciferase (luc) and used to measure cell viability/survival with the BrightGlo reagent (Promega # E2650). Human T cells were isolated from peripheral blood mononuclear cells (PBMCs) of healthy human donors using the pan T cell isolation kit (Miltenyi #130-096-535) according to manufacturer’s recommended protocol. The isolated T-cells were further expanded using Human T-Activator CD3/CD28 Dynabeads (Gibco #11132D) for nine days, then debeaded magnetically and stored as viable frozen aliquots in liquid nitrogen tank. The expanded T cells were used as effector T cells in RTCC assays where they were thawed from frozen aliquots, counted, and used immediately at an Effector:Target (E:T) cell ratio of 3:1 or as indicated. Target cells were plated at 30,000 cells per well in a 96 well plate (Costar # 3904) together with thawed T-cells and a serial dilution of various BCMAxCD3 BisAbs, all in media containing RPMI/1640, 10% FBS, 2mM L-glutamine, 0.1 mM non-essential amino acids, 1mM Sodium pyruvate, 10mM HEPES, and 0.055mM 2-mercaptoethanol (Gibco # 22400089, 16140, 25030-081, 11140-050, 11360-070, 15630-080, and 21985-023 respectively). These treatments were compared in the presence or absence of either of two GSIs: DAPT [1.0 μM] (Enzo Life Sciences, # ALX-270-416-M005), or LY-411,575 [0.1 μM]. The assay was incubated at 37°C and 5% CO2 for 20-24 hours, followed by measurements of target cell viability (BrightGlo, Promega # E2650), following vendor supplied protocols. In addition, target MM cell lines were treated with and without GSIs in the absence of T cells to determine the effect of GSIs on cell viability (72h treatment) and mBCMA density (overnight treatment). Cell viability was quantified using BrightGlo, whereas mBCMA density was determined by FC analysis, as described earlier for KMS11 cells.

**FC-based RTCC/TDCC assay**

The effector cells were incubated with target MM cell lines or primary MM cells at indicated E:T ratios (1:1, 3:1, 5:1, 6:1 or 10:1) to mimic sub-optimal assay conditions in the presence of serial dilutions of controlxCD3 ER79 or BCMAxCD3 (ER26, BU76, BQ76, PL33) BisAbs, with or without sBCMA, or indicated GSIs (2 nM LY-411575, 1 μM DAPT unless otherwise mentioned). Co-cultures were maintained in a final volume of 100μl culture media at 37°C with 5% CO_2_. For long time incubation, equal volume of prewarmed complete culture media was added on day 4. After 4h, 1d, 4d or 7d co-incubation, Abs were used to stain CD3^+^ T cells and CD138^+^ MM cells followed by FC-based investigation. The number of viable MM cells was determined by 123count eBeads™ Counting Beads (Thermo Fisher Scientific). The lysis of viable MM cells (means ± SDs) was calculated using the following formula:

% Target cell lysis = 100 - (viable cell number of indicated treatment group × 100/viable cell number at start).

After 1d incubation, the quantitative FC-based analysis using Annexin V and LIVE/DEAD™ Fixable Aqua Dead Cell Stain Kit (Thermo Fisher Scientific) was also applied to evaluate CD138^+^ cell elimination.

BMMCs and PBMCs derived from MM patients were mixed with the same cell numbers and used to estimate autologous patient MM cell lysis by indicated BCMAxCD3 vs control BisAbs in the presence or absence of indicated GSIs. Sample (patient BMMCs and PBMCs) viability was > 98%, as confirmed by Annexin V-/Aqua-negativity using Annexin V and LIVE/DEAD™ Fixable Aqua Dead Cell Stain Kit (Thermo Fisher Scientific). BMMCs, pre-treated with or without GSIs (2 nM LY411575 or 1 μM DAPT) were co-cultured in the presence of indicated BCMAxCD3 vs control BisAbs, in 96-well U-bottom plates for 1d and 4d (when GSIs were co-treated with BisAbs). Surviving CD138^+^ patient cells were enumerated by quantitative FC analysis of CD138^+^ cells in the presence of Flow-Count 123 beads (Thermo Fisher Scientific) and LIVE/DEAD™ Fixable Aqua Dead Cell Stain Kit (Thermo Fisher Scientific). The percentage of CD138^+^ patient cell lysis (depletion) was calculated using the following formula: % lysis of CD138^+^ patient cells = [1- (number of surviving CD138^+^ cells/number of CD138^+^ cells at start] × 100%.

**Degranulation assay (CD107a mobilization)**

PBMCs or T-cells were incubated in 96-well plates (100,000 cells/well) together with indicated MM cell lines or BMMCs from MM patients. Co-cultures were maintained in a final volume of 100μl culture media (5% Human Serum in LGM-3 with 2 ng/ml IL-2) for 6h at 37°C and 5% CO2 with Protein Transport Inhibitors (BFA and Monensin). CD107a staining was done during cell stimulation by the addition of a fluorescent anti-CD107a Ab during the last hour of the co-culture. The degranulation activity was evaluated by FC-based analysis and determined as the percentage of CD107a^+^ CD8^+^ T-cells.

**Thy1-cytokine expression analysis**

For intracellular cytokine staining, Protein Transport Inhibitors (BFA and Monensin) were first added immediately after effector cells and MM cells were co-cultured for 6h. The cells were then collected, permeabilized, and fixed by Cytofix/Cytoperm kit (BD) according to the manufacturer’s instruction. They were stained with anti- IFN-γ, anti-IL-2, and anti-TNF-α antibodies followed by FC analysis.

**Quantitative FC analysis for MM-PBMC/T-co-cultures**

On day 0, 1, 3, and 7 of co-incubation, the cells were collected also for various memory T cell subtype analysis. The subsets of memory T cells were identified by expression of CD45RA and CD62L on the T cell surface, including naïve, central memory (CM), effector memory (EM), and terminal effector memory RA (TEMRA) in CD4^+^ and CD8^+^ T cells gated by the expression of CD45RA and CD62L (naïve, CD45RA^+^CD62L^+^; CM, CD45RA^-^CD62L^+^; EM, CD45RA^-^CD62L^-^; terminal effector, CD45RA^+^CD62L^-^).^4^ The cell mixture in co-cultures was first stained with Abs against CD3, CD4, CD8, CD25, and CD138 surface markers. The cells were then fixed and permeabilized, followed by intracellular staining with Abs against FOXP3, IL-10, and TGF-β1. The percentages of IL-10 and TGF-β1 expression as well as Treg (CD25^+^FOXP3^+^) in CD4 T cells were also determined as previously reported^5-7^.

MM cell lines, BMMCs, and PBMCs/T-cells, alone or in co-cultures, in the presence or absence of GSIs, with or without indicated BisAbs, following indicated treatment time periods were first stained with Live/Dead Fixable Aqua Dead Cell Staining Kit (no. L34957, Thermo Fisher Scientific) and Annexin V PE conjugate (no. 640908, BioLegend), in conjunction with plasma cell and T cell subset markers according to manufacturer’s instruction.

The Wilcoxon signed rank test was used to evaluate the statistical significance of the differences observed between mean fluorescence intensity (MFI) levels of indicated T-lymphocytes in co-cultures, whereas the Mann–Whitney U and the Kruskal–Wallis tests were used to estimate the statistical significance of differences observed between two or more groups, respectively. Correlation studies were performed using the Pearson test. Survival curves were plotted according to the Kaplan–Meier method and compared using the log-rank test.

**In vivo adoptive transfer (AdT)-NSG mouse model of MM**

***Cell lines***

The luciferase cell line was created from stably transfected pooled clones at NIBR Emeryville. The current NIBRI stock of KMS11-Luc used for these studies was initially received from NIBR Emeryville, expanded and viably frozen at NIBRI, Cambridge. All animal studies were performed under approval by the Novartis Institutes for BioMedical Research Institutional Animal Care and Use Committee and in compliance with the Guide for the Care and Use of Laboratory Animals.

***In vivo assessment of soluble BCMA in the serum of tumor bearing mice***

5x10^6^ KMS11-Luc cells were implanted subcutaneously. Prior to treatment, animals were bled for baseline levels of sBCMA. When tumor volume (TV) reached an average of ~325 mm^3^ (d17), mice were randomized and treated with single dose of LY411575 (3.0 and 10 mg/kg) or vehicle control. All treatments were administered PO at 10 mL/kg. Serial whole blood collections were done at 1, 3, 8, and 24 hours post dose. Samples were processed for serum and stored in a -20C freezer until evaluated for levels of sBCMA.

***Detection of human sBCMA in mouse serum samples***

Approximately 30-50µL of whole blood was collected in serum tubes (no additive, microvette CB 300, Sarstedt) via tail snip for serial sampling or cardiac puncture for terminal procedures and processed to serum. The serum was stored at -20°C for batched analysis of sBCMA levels by ELISA. Detection of soluble human BCMA was carried out by DUOSet ELISA – human BCMA assay (R&D Systems, DY193). Data was captured on a SpectraMax multi-mode plate reader (Molecular Devices) and quantified using SoftMax Pro (Molecular Devices). Standard and serum samples were run in duplicate, and extrapolated values were adjusted for dilution factor. Pre-bleeds were collected on D0 prior to dosing. Data analysis was performed using GraphPad Prism software (GraphPad v8.1.2, CA) using four-parameter data extrapolation.  Fold change was calculated by taking the observed sBCMA concentration of each individual animal at the specific time point and dividing by their respective pre-bleed value (i.e., observed sBCMA concentration/pre-bleed concentration). Percent inhibition was calculated with the following formula of ((average of all untreated controls – average test sample)/average control) x 100.

***MM Anti-tumor activity of PL33 and LY411575 in the adoptive transfer model***

Female NOD/SCID/GAMMA (NSG) mice ~6-8 weeks old were purchased from The Jackson Laboratory and allowed to acclimate for at least 3 days prior to manipulation. Following acclimation, the mice received an adoptive transfer of 15x10^6^ freshly thawed PBMC IV into the lateral tail vein. Five days following the adoptive transfer, KMS11-luc cells were implanted subcutaneously into the right flank at an inoculum of 5x10^6^ cells/mouse. Animals were monitored once weekly for body weight and tumor volume prior to study initiation and twice weekly following study initiation. Tumor length and width were captured manually by a caliper and tumor volume was calculated using the formula TV (mm^3^) = π x Length x (width)^2^/6. When the average tumor volume reached ~ 200 mm^3^ (198 ± 47 mm^3^), mice were randomized into their respective groups: control (tumor only and Tumor + PBMC) or treatment, either LY411575, PL33, or a combination of both. The BCMAxCD3 bispecific antibody, PL033, was formulated in 0.2nM citrate, 0.01M NaCl, 0.05M sucrose, 0.02% Tween80 in Hyclone water for injection to its final concentration of 30 mg/mL or 10 mg/mL. The gamma secretase inhibitor, LY411575, was formulated in 10% Ethanol, 60% PEG400, and 20% PG in 0.4% MC to its final concentration of 3 mg/mL. All treatments were administered at 3 mL/kg according to individual mouse body weights. LY411575 was administered for 2 consecutive days by oral gavage: 24 hours prior to and co-administered with PL33. PL33 was administered as a single IV injection into the lateral tail vein 2 hours post the second dose of LY411575.

***Determination of anti-tumor activity and change in body weight***

Anti-tumor activity was determined by percent change in tumor burden (TB) versus control (%ΔT/ΔC) using the following formulas: if ΔTB≥ 0, 100 X ΔTB treatment, time /ΔTB control group, time, if ΔTB< 0, percent regression (%∆T/T0) was used (-1 X (100 X (TB final-TB initial/ TB initial), TB initial is the tumor burden on the day of treatment initiation. %ΔT/ΔC values <42% were considered to have anti-tumor activity. Anti-tumor activity of each group was compared to an untreated control group that received tumor implant and donor PBMCs but no treatment (tumor + AdT). The tumor only group is included to meter the allogeneic response observed with untreated control. This study had minimal allogeneic response. A secondary read for efficacy was scored by tumor growth delay, defined as Time to Endpoint (TTE) determined by Kaplan-Meier analysis, comparing median time to endpoint. Animals remained on study until each reached individual endpoints, defined by tumor volume > 1200mm^3^, tumor ulceration, body weight loss >20% or poor body condition. The Time to endpoint graph was generated in Prism whereas the survival statistics were analyzed using SigmaPlot. Percent body weight change was determined using the formula: 100 X ((BW time – BW initial)/BW initial). Individual response criteria were also evaluated and scored as either Complete Response (CR), no detectable tumor at time of last measurement; Partial Response (PR), tumor volume less than baseline measurement at any time point followed by regrowth; or No Response (NR), tumor continues to increase over baseline measurement throughout the study. The institutional animal care and use committee approved of all in-vivo experiments.

***Statistical analyses***

Anti-tumor activity was evaluated using 1-way analysis of variance (ANOVA) with the Tukey post test, or an unpaired t-test with assumed similar variance using GraphPad Prism Software, Version. 9.2.0. Kaplan-Meier Survival statistics were analyzed using Log-rank test with all pairwise multiple comparison ad hoc (Holm-Sidak method) using SigmaPlot v.14.0. Results with a P value of ≤0.05 were considered significant.

**References**

1. Yu T, et al. VIS832, a novel CD138-targeting monoclonal antibody, potently induces killing of human multiple myeloma and further synergizes with IMiDs or bortezomib in vitro and in vivo. *Blood Cancer J.* 2020;10:110.

2. Lin L, et al. Preclinical evaluation of CD8+ anti-BCMA mRNA CAR T cells for treatment of multiple myeloma. *Leukemia.* 2021;35:752-63.

3. Xing L, et al. A novel BCMA PBD-ADC with ATM/ATR/WEE1 inhibitors or bortezomib induce synergistic lethality in multiple myeloma. *Leukemia.* 2020;34:2150-62.

4. Cho SF, et al. The immunomodulatory drugs lenalidomide and pomalidomide enhance the potency of AMG 701 in multiple myeloma preclinical models. *Blood Adv.* 2020;4:4195-207.

5. Feng X, et al. Targeting CD38 suppresses induction and function of T regulatory cells to mitigate immunosuppression in multiple myeloma. *Clin Cancer Res.* 2017;23:4290-300.

6. Zhang L, et al. Regulatory B cell-myeloma cell interaction confers immunosuppression and promotes their survival in the bone marrow milieu. *Blood Cancer J.* 2017;7:e547.

7. Tai YT, et al. APRIL signaling via TACI mediates immunosuppression by T regulatory cells in multiple myeloma: therapeutic implications. *Leukemia.* 2019;33:426-38.

**Supplementary results**

**
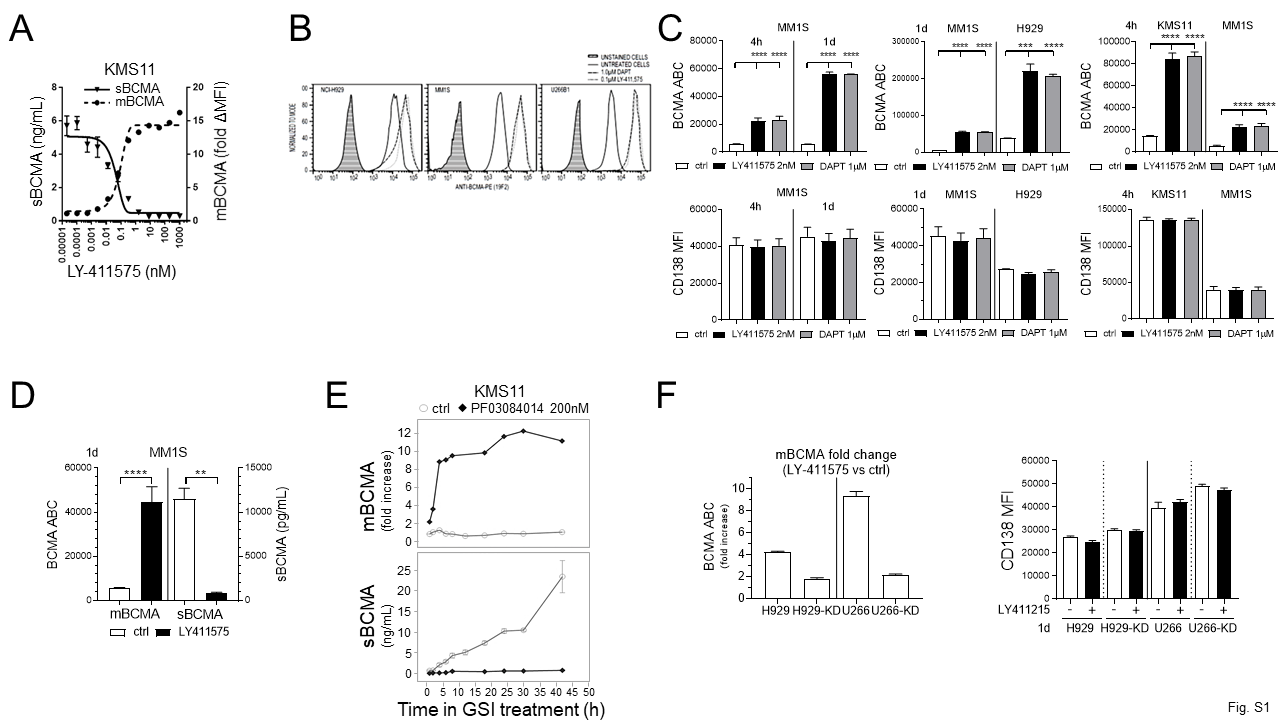
**

**Supplementary Fig. S1 GSIs selectively increased mBCMA but not mCD138 levels, associated with shed BCMA (sBCMA) depletion in the MM cells and paired culture supernatants.**

Indicated MM cell lines (**A**, **E**: KMS11; **B-C**, **F**: H929; **B**: U266; **B**, **C**-**D**: MM1S) were treated with indicated GSI (**A**-**D** and **F**, LY411575; **B**-**C**, DAPT; **E**, PF03084014) for indicated time periods (**A**-**D** and **F**, 1d; **C**, 4h; **E**, up to 43h) followed by quantitative FC analysis to determine BCMA MFI (**A**-**B**, **E**) and BCMA ABC (**C**, upper panels; **D**, left y-axis); **F**, left). CD138 MFI is also shown in **C** (lower panels) and **F** (right). Shown are also sBCMA levels in the paired cell culture supernatants (**A**, left y-axis; **D**, right y-axis; **E**, lower panel). (**E**) KMS11 cells were treated with 200 nM PF03084014 for up to 42h. (**F**) Parental and BCMA-KD paired H929 and U266 cell lines were incubated with LY411215 (2 nM) (**Fig. 1E**). Three independent experiments were done with each treatment condition in triplicate. Data are presented as means ± standard deviations (SDs) (error bars). **P* < .05, ***P* < .01, ****P* < .001, *****P* < .0001

**
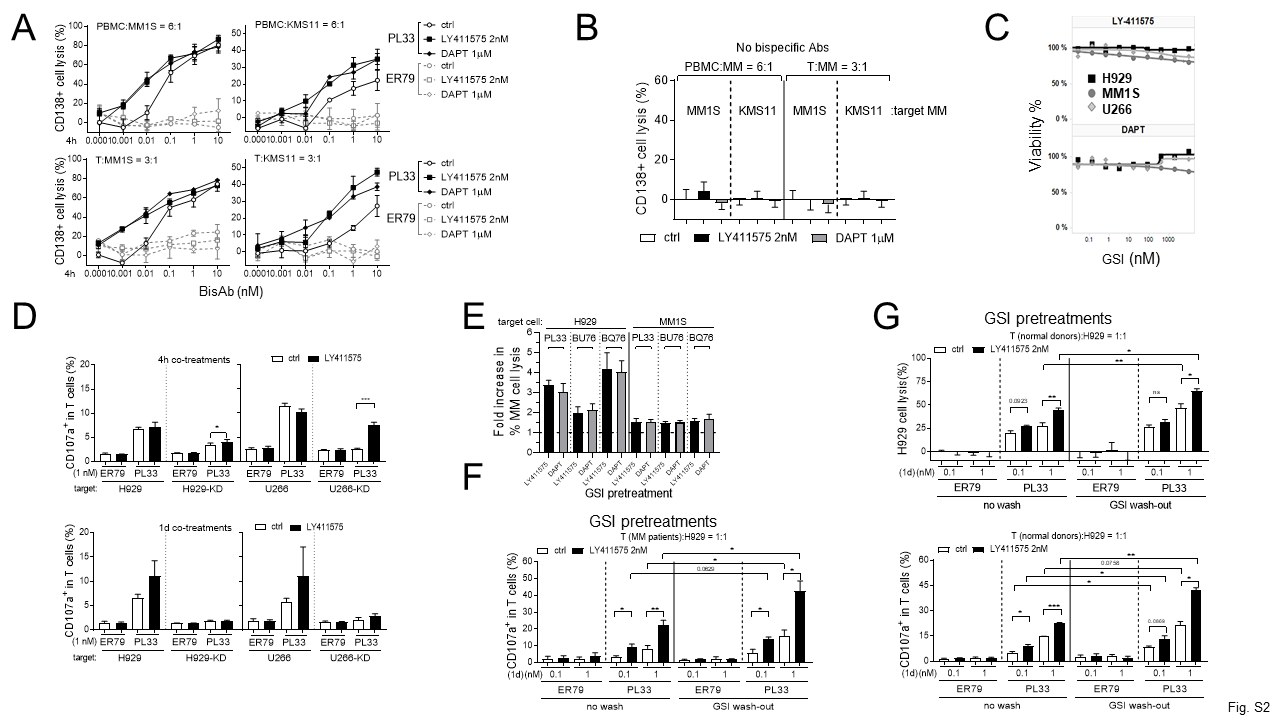
Supplementary Fig. S2 GSIs decreased inhibition of sBCMA in MM cell lysis induced by BCMAxCD3 bispecific Ab, without impacting viability of MM cells, alone or in the presence of effector cells.**

BCMAxCD3 (**A**, **D**-**G**, PL33; **E**, BU76 and BQ76) or ER79 (ControlxCD3) BisAbs (1 nM or indicated concentrations) were added for 4h (**A**, **E**) or 1d (**D**, lower panel; **F**-**G**) in the co-culture of MM target (**A**-**B**: KMS11; **A**, MM1S; **D**-**G**: H929) and T effector cells, in the presence or absence of indicated GSIs (**A**, **D**-**G**: 2 nM LY411575; **A**, **E**: 1 μM DAPT). T cell-dependent MM cell lysis was determined by FC-based (**A**-**B**; **E**; **G**, upper panel) analysis. (**B**) No BisAbs were added in co-cultures of MM1S or KMS11 target cells with effector cells for 1d in the presence or absence of LY411575 or DAPT. (**C**) Viability of 3 indicated MM cell lines following 72h treatment with serial dilutions of 2 indicated GSIs (0-1000 nM). (**D**, **F**-**G**) CD107a surface expression on T effector cells from MM patients (**D**, as in **Fig. 2C**; **F**, as in **Fig. 2E**) or normal donors (**G**, n=3) was determined by quantitative FC analysis. Independent experiments using effector cells from multiple individuals (3 normal donors in **A**-**B**, **G**; MM patients in **D** (n = 5), **E** (n = 8), **F** (n = 5)) were done with each treatment condition in triplicate. Data are presented as means + SDs (error bars). *P < .05; **P < .01; ***P < .001; ****P < .0001; ns, not significant

**
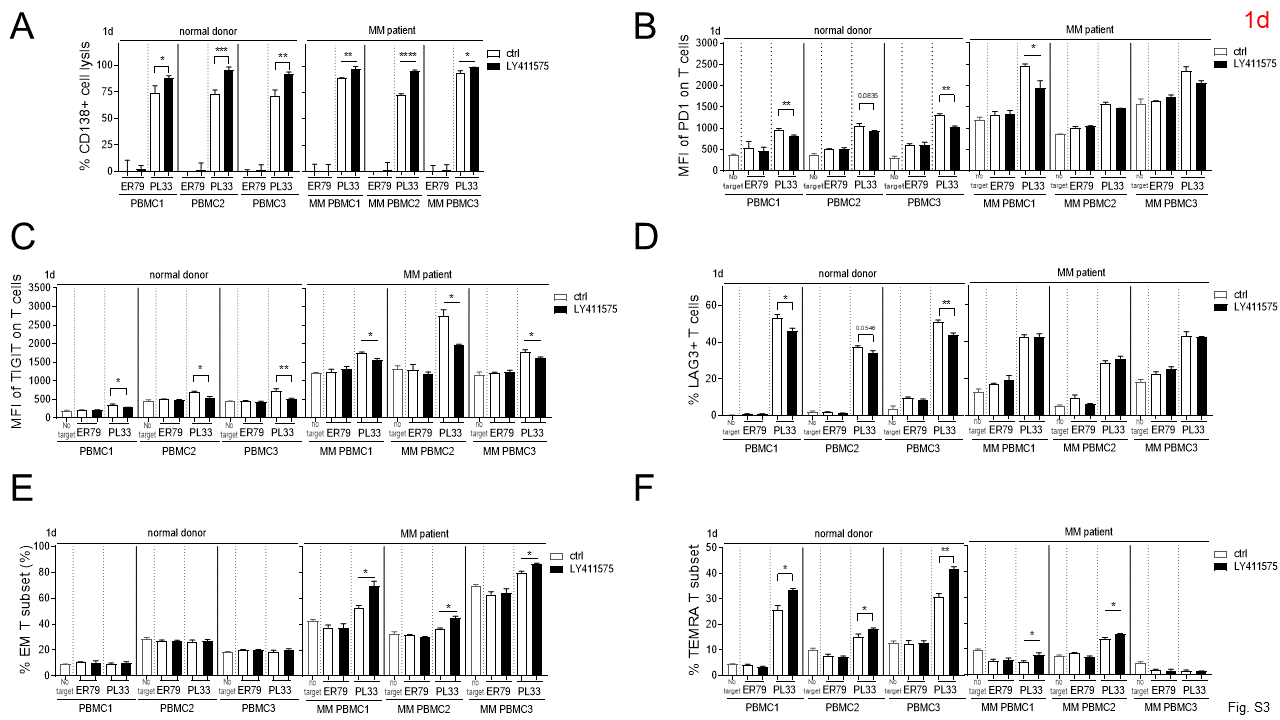
Supplementary Fig. S3 GSI co-treatment did not further induce expression of T cell checkpoint markers and alter T cell differentiation by PL33 in the MM-PBMC co-cultures in 1d ex vivo MM-PBMC co-cultures.**

PBMCs from normal donors (n = 3) or MM patients (n = 3) were co-cultured with MM1S target cells (E:T = 5:1) for 1d in the presence of ER79 or PL33 (10 nM), with or without LY411575 (2 nM). Quantitative FC analysis was used to determined % CD138^+^ cell lysis (**A**), levels of indicated checkpoint molecules on T cells (**B**, PD1; **C**, TIGIT; **D**, LAG3), and % differentiated T-cell subtypes including effector memory (EM, CD45RA−CD62L−) (**E**) and terminal effector memory RA (TEMRA, CD45RA^+^CD62L^−^) (**F**). All co-culture experiments were done in quadruplicate at each condition. Data are presented as means ± SDs (error bars). **P* < .05; ***P* < .01; ****P* < .001; *****P* < .0001; ns, not significant

**
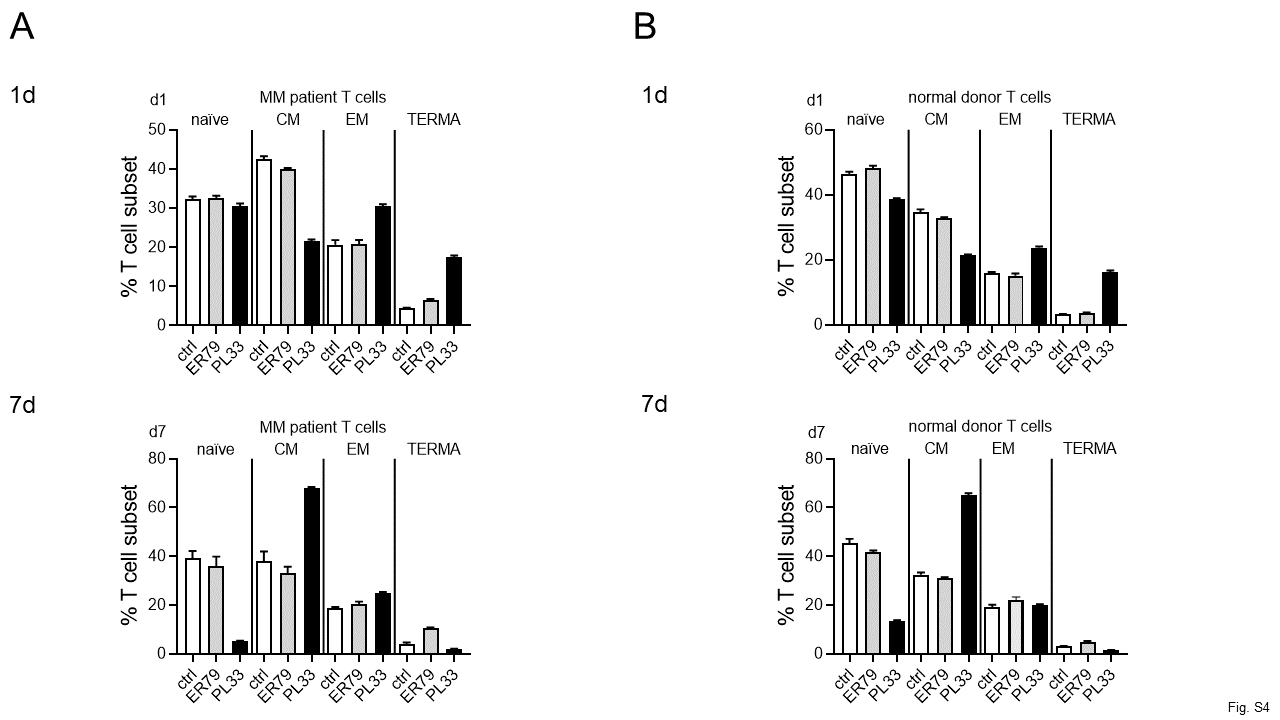
Supplementary Fig. S4 PL33 induced T cell differentiation with memory phenotypes in MM1S-T co-cultures for 7d.**

MM1S cells were co-cultured with T cells from MM patients (**A**) or normal donors (**B**) (n = 3 each) (E:T = 1:1) in the presence of PL33 or ER79 for 1d and 7d followed by quantitative FC analysis to determine indicated T cell subsets (naïve: CD45RA^+^CD62L^+^; CM, 45RA^−^CD62L^+^; EM, CD45RA^−^CD62L^−^; TEMRA, CD45RA^+^CD62L^−^).
